# Commentary: Visual Feedback of Tongue Movement for Novel Speech Sound Learning

**DOI:** 10.3389/fnhum.2016.00662

**Published:** 2016-12-23

**Authors:** Marcelo L. Berthier, Ignacio Moreno-Torres

**Affiliations:** ^1^Cognitive Neurology and Aphasia Unit and Cathedra ARPA of Aphasia, Centro de Investigaciones Médico-Sanitarias, Instituto de Investigación Biomédica de Málaga, University of MalagaMalaga, Spain; ^2^Department of Spanish Language, University of MalagaMalaga, Spain

**Keywords:** visual feedback, auditory feedback, tongue movements, speech learning, neuroimaging, cholinergic systems

Access to visual information during speech is useful for verbal communication because it enhances auditory perception, especially in noise (Sumby and Pollack, 1954). Until recently, visual feedback of the speaker's face was possible only for the visible articulators (the lips), but new computer-assisted pronunciation training (CAPT) systems have created a kind of “transparent” oral cavity that permits us to look at our own tongue movements during articulation.

The use of CAPT systems is a clever way to integrate visual feedback with auditory feedback. This has been exploited by Katz and Mehta (2015) (hereafter K & M) to strengthen learning of novel speech sounds. Using this emerging methodology (*Opti-Speech* visual feedback system—Katz et al., 2014), they trained five college-age subjects to learn a novel consonant in the /ɑCɑ/ context. Before training, the stimulus was produced by one of the investigators (SM) three times together with an explanation on how to position the tongue inside the mouth while reproducing the phoneme. The learned consonant was a sound not attested as a phoneme among the world's languages. The stimuli were elicited in blocks of 10 /ɑCɑ/ productions using a single-case ABA design (pre-training, training, post-training), with visual feedback only in the training phase. In their study, K & M presented the listeners with a visual representation of internal (hidden to vision) articulators to enhance perception and eventually to finely tune articulatory tongue movements. Five sensors were positioned on the tongue of the learners so as to provide them with online visual feedback of the hidden articulators thus allowing tongue reading. Despite receiving detailed verbal instructions, all subjects did poorly at baseline assessment; their accuracy improved during the visual feedback training and gains were maintained in a follow-up examination (both *p* < 0.001 relative to baseline). The effect size of training for each subject was >90% (highly effective). Analysis of acoustic and spectral parameters suggested increased production consistency after training.

Nevertheless, K & M's findings should be interpreted with caution as the study had some methodological drawbacks, which limit firm conclusions on the reported effectiveness of the training procedure. These include the small sample size, the uncontrolled nature of the study, the fact that not all participants completed the initial protocol, the use of a novel consonant (not attested as a phoneme in any natural language), and the unreliable replicability of target phoneme produced by the experimenter. These factors need to be controlled in future studies to ascertain whether motor learning of tongue movements with the support of 3D information is useful to learn speech sound in real life situations (L2 learning and speech-language rehabilitation). In any case, K & M's study represents a step forward in the use of multimodal information for second language learning in healthy subjects and for treating abnormal speech in different conditions (stuttering, apraxia of speech, foreign accent syndrome—FAS-).

K & M also outlined the pathways providing external visual feedback and internal feedback using the neurocomputational ACT model of speech production and perception (Kröger and Kannampuzha, 2008). The key role of cortical areas mediating body awareness (insula) and reward during behavioral training (lateral premotor cortex) has also been discussed. In a recent study using real-time functional magnetic resonance imaging (rtfMRI) during visual neurofeedback, Ninaus et al. (2013) found activation of bilateral anterior insular cortex (AIC), anterior cingulate cortex (ACC), and supplementary motor and dorsomedial and lateral prefrontal areas. Gaining further knowledge on the multiple components of these networks is particularly telling for intensive learning training and for therapeutic purposes because using rtfMRI during neurofeedback humans can learn to voluntarily self-regulate brain activity in circumscribed cortical areas (Caria et al., 2007; Rota et al., 2009). The AIC is one such key circumscribed area susceptible to be voluntarily self-controlled (Ninaus et al., 2013). By acting as a multimodal integration hub, the AIC is in an ideal position for coordinating the activity of several networks devoted to modulate multisensory information (visual, auditory-motor, tactile, somatosensory, Ackermann and Riecker, 2010; Moreno-Torres et al., 2013) involved in feedback of tongue movements during the speech production. In addition, note that healthy subjects activate both AIC during overt speech (Ackermann and Riecker, 2010) and that the AIC together with the left frontal operculum are co-activated during learning foreign sounds (Ventura-Campos et al., 2013). The AIC also contains the sensory-motor maps that code the subjective feeling of our own movements (body awareness; Critchley et al., 2004; Craig, 2009). Acting conjointly with the ACC, the AIC is engaged in speech initiation (Goldberg, 1985), cognitive control, goal-directed attention, and error detection (Nelson et al., 2010) during the execution of effortful tasks (Engström et al., 2015).

Cue detection and attentional control involved in visual feedback probably depend upon the activation of nicotinic acetylcholine receptors (Demeter and Sarter, 2013) in the AIC and ACC (Picard et al., 2013). In support of this view, learning deficits induced by experimentally-induced insular lesions are reverted by stimulation of cholinergic neurotransmission (Russell et al., 1994). Future studies will examine the modulation of neurochemistry with non-invasive brain stimulation (Fiori et al., 2014), drugs (Berthier and Pulvermüller, 2011), or both to ascertain whether the results of novel speech sound learning reported by K & M using visual feedback of tongue movements can be accelerated and maintained in the long-term. Preliminary evidence of the beneficial effect of combining audio-visual feedback training and cholinergic stimulation on reverting FAS has been demonstrated in a single patient (Berthier et al., 2009; Moreno-Torres et al., 2013). Importantly, the almost complete return to native accent in this case of FAS was associated with partial or total normalization of metabolic activity in several regions of the speech production network, especially in the left AIC and ACC (Figure 1).

**Figure 1:**
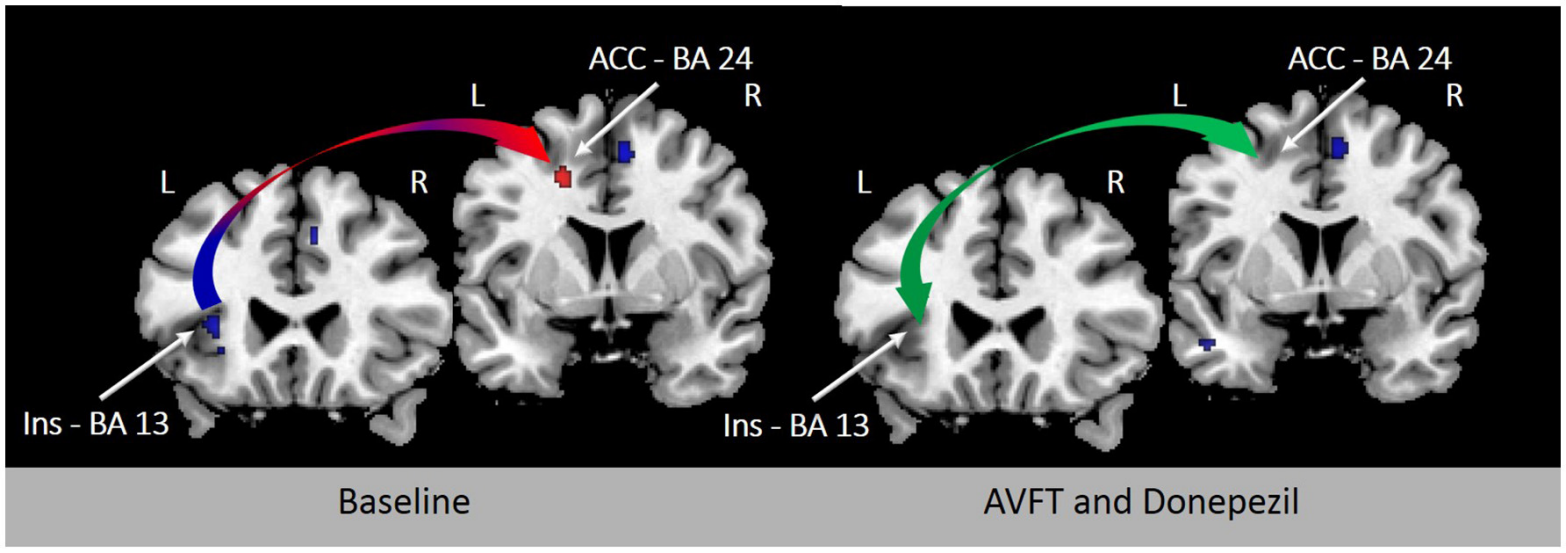
**Resting [^18^F]-fluorodeoxyglucose positron emission tomography (^18^FDG-PET) before and after treatment in a 44-year-old woman with chronic post-stroke foreign accent syndrome (FAS)**. Rates of metabolic activity in this single patient were compared with 18 healthy control subjects (female/male: 11/7; mean age ± SD: 56.6 ± 5.7 years; age range: 47–60 years). The left panel shows T1-weighted coronal sections depicting significant hypometabolism in the left deep frontal operculum and dorsal anterior insula (Talairach and Tournoux peak coordinates: *x* − 36 *y* 14 *z* 16) (blue arrow) with significantly increased compensatory metabolic activity in the left ACC (Talairach and Tournoux peak coordinates: *x* −18 *y* 8 *z* 40) (red arrow) before treatment (Moreno-Torres et al., 2013). Clusters in baseline ^18^FDG-PET were significantly larger with family-wise error correction (*p* < 0.05) in comparison to healthy controls. Deficient phonetic error awareness and monitoring responsible from FAS in this patient were treated with audiovisual feedback training (visually-guided using Praat—(Boersma and Weenink, 2010)—and adjusting patient's emissions to F0 contours of sentence-models) alone and in combination with a cholinergic drug (donepezil, 5 mg/day). An almost complete return of accent to its premorbid characteristics was observed after combined treatment and these changes correlated with normalization of metabolic activity in the left AIC and ACC (green arrow; right panel; Berthier et al., 2009). BA indicates Brodmann area.

In summary, the results of K & M open new important avenues for future research on the role of visual feedback in learning new phonemes. Further refinement of these methodologies coupled with a better understanding of the neural and chemical mechanisms implicated in tuning tongue movements with audio-visual feedback to learn new phonemes represent a key area of enquire in the neuroscience of speech.

## Author contributions

The two authors (MB and IM) have made substantial, direct and intelectual contribution to the work, and approved it for publication. MB and IM drafted the article and revised it critically for important intelectual content.

## Funding

This research was partly supported by a grant from the Spanish Ministeriode Economía e Innovación to the second author (FFI2015-68498P).

### Conflict of interest statement

The authors declare that the research was conducted in the absence of any commercial or financial relationships that could be construed as a potential conflict of interest.
